# An Effective Cuckoo Search Algorithm for Node Localization in Wireless Sensor Network

**DOI:** 10.3390/s16091390

**Published:** 2016-08-31

**Authors:** Jing Cheng, Linyuan Xia

**Affiliations:** Guangdong Key Laboratory for Urbanization and Geo-simulation, School of Geography and Planning, Sun Yat-Sen University, Guangzhou 510275, China; chengjing921@126.com

**Keywords:** wireless sensor network, localization, cuckoo search algorithm, average localization error, convergence rate

## Abstract

Localization is an essential requirement in the increasing prevalence of wireless sensor network (WSN) applications. Reducing the computational complexity, communication overhead in WSN localization is of paramount importance in order to prolong the lifetime of the energy-limited sensor nodes and improve localization performance. This paper proposes an effective Cuckoo Search (CS) algorithm for node localization. Based on the modification of step size, this approach enables the population to approach global optimal solution rapidly, and the fitness of each solution is employed to build mutation probability for avoiding local convergence. Further, the approach restricts the population in the certain range so that it can prevent the energy consumption caused by insignificant search. Extensive experiments were conducted to study the effects of parameters like anchor density, node density and communication range on the proposed algorithm with respect to average localization error and localization success ratio. In addition, a comparative study was conducted to realize the same localization task using the same network deployment. Experimental results prove that the proposed CS algorithm can not only increase convergence rate but also reduce average localization error compared with standard CS algorithm and Particle Swarm Optimization (PSO) algorithm.

## 1. Introduction

A wireless sensor network (WSN) is a self-organization network composed of a large number of small-size, low-cost sensor nodes which can monitor physical or environmental condition [1]. Recent advances of micro-electro-mechanical systems (MEMS) technology and wireless communication have propelled WSN applied to a variety of fields such as health monitoring [2], transportation management [3], business and home automation [4], global-scale wildlife [5], forest fire and environmental monitoring [6,7]. In almost all the above applications, on no account can we ignore the value of location information of sensor nodes, because it is extremely hard to distinguish or utilize the monitored information without location information. Besides that, geographic routing can save significant energy by eliminating the need for route discovery [8]. Therefore, it is highly significant to locate sensor nodes accurately.

Traditionally, each sensor node can be localized by using the BeiDou Navigation Satellite System (BDS) or Global Positioning System (GPS). However, practical considerations such as cost, power consumption and the volume of BDS/GPS receivers make it impossible for the use of BDS/GPS on each sensor node, especially for wide-range WSN. For example, sensor nodes are typically powered by small batteries, which generally cannot be easily changed or recharged. In addition, WSN is commonly deployed in the urban canyon or indoors where the transmission of the satellite signals is adversely blocked [9].

**Figure 1:**
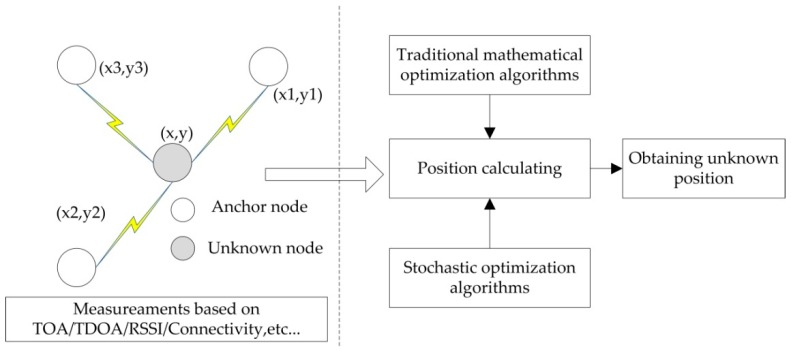
Node localization process in WSN.

In recent years, more and more researchers tried to overcome the drawbacks by proposing various localization methods. Generally WSN node localization process can be illustrated using Figure 1. In a two-dimensional (2-D) field, it is assumed that the unknown node’s coordinate is (x, y) which needs to be positioned, anchor node’s coordinates are (x1, y1), (x2, y2), (x3, y3). The unknown node will be localizable if there are three (or more) anchors, which have prior knowledge of their coordinates within its communication range.

As presented in Figure 1, the localization process consists of two stages: measurements based on inter-sensor distance, angle measurements or connectivity, such as Time of Arrival (TOA), Time Difference of Arrival (TDOA), Angle of Arrival (AOA), Received Signal Strength Indication (RSSI), etc. [10]; then calculating position of unknown nodes whose coordinates are unknown either by traditional mathematical optimization methods solving a set of simultaneous equations, or by using stochastic optimization algorithms that minimize localization error. Whatever measuring technology is adopted, in actual scenario measuring data is always disturbed by some noise, which will make localization results dissatisfactory [11]. Hence a lot of literatures focus on overcoming the problem.

A detailed survey of node localization in WSN has been reported in the literature [12,13]. Niculescu et al. [14] proposed the efficient Ad Hoc Positioning System (APS) that extends the capabilities of GPS to non-GPS enabled nodes in a hop by hop fashion in an ad hoc network where only a limited fraction of nodes have self-location capability. Anchors flood their location information to all the neighboring nodes and each unknown node estimates its own location by performing distance measurement from three or more anchors. To prevent error accumulation, Rabaey et al. [15] introduced a two-phase approach using connectivity between nodes for initial position estimates and only the distances to one-hop neighbors are considered for position refinement. This approach reduces insensitivity to range errors on some extent. Doherty et al. [16] reported an efficient second-order cone method to solve the localization problem by relaxing the proximity constraints between nodes. This method requires anchors deployed around the perimeter of the network, otherwise the position estimation of unknown nodes will tend to interior, yielding highly inaccurate results. Based on [16], other researchers [17] studied a semi-definite programming (SDP) relaxation method to solve sensor network localization problem using incomplete and inaccurate distance information. This method performs well even in highly noisy environments. In 2014, Simonetto et al. [18] presented a class of convex relaxations based on a maximum likelihood (ML) formulation, which derived a computational efficient edge-based version of this ML convex relaxation class and designed a distributed algorithm that enable sensor nodes to solve these edge-based convex programs locally by communicating only with their close neighbors. Employing this version of convex relaxations to message the original non-convex formulation can offer a powerful handle on computing accurate solutions. Shang et al. [19] introduced a localization algorithm MDS-MAP (P), which worked in a distributed mode by using relative maps. This algorithm keeps better performance than basic MDS-MAP algorithm under the uniform layouts or irregularly-shaped networks, particularly when the number of anchors is small. However, it needs a high consumption of battery power for each sensor to construct relative maps. Although these traditional mathematical optimization techniques work well, they require enormous computational efforts and communication overhead, which grow exponentially as the problem size increases.

In recent years meta-heuristic algorithms gradually play an important role in engineering optimization [20,21]. This is attributed to the fact that they require moderate memory and computational efforts and more importantly can provide better results in comparison with traditional mathematical optimization methods. WSN node localization is often formulated as a multi-dimensional optimization problem. A survey of bio-inspired node localization can be found in [22,23]. To meet the localization requirement of large scale WSN, Yun et al. [24] reported two intelligent range-free WSN localization schemes, which utilize received signal strength (RSS) from the anchor nodes. First, the edge weight of each anchor node is considered to be separately and combined to compute the location of sensor nodes. The edge weights are modeled by the fuzzy logic system (FLS) and optimized by the genetic algorithm (GA). Second, localization is considered as a single problem and the entire sensor location mapping from the anchor node signals is approximated by a neural network (NN). To address the flip ambiguity problem, Kannan et al. [25] introduced a two phase simulated annealing (SA) based localization algorithm, which first obtains an accurate estimate of location, then if some nodes have flip ambiguity problem, optimization is performed based on neighborhood information of nodes and moved to the correct position. As a promising population-based optimization algorithm, Particle Swarm Optimization (PSO) algorithm was first developed by James and Russell [26] in 1995 and has been successfully applied in various fields. For example, Chih [27] presented extensive literature review on PSO and introduced a novel self-adaptive check and repair operator (SACRO) based on BPSO-TVAC and CBPSO-TVAC [28] to solve the multidimensional knapsack problem. Experimental results show that the proposed algorithm is more competitive and robust than the traditional CRO. To improve location accuracy, Gopakumar et al. [29] devised a WSN localization scheme using Particle Swarm Optimization (PSO) algorithm. However, it is likely to get trapped in local optimal that leads to pre-matured convergence. Biogeography based optimization (BBO) and its variants [30], hybrid algorithms such as bacterial foraging approach (BFA) and GA [31], PSO and BFA [32], PSO and DE [33], GA and SA [34], have been also developed in order to improve localization accuracy or convergence speed. However, the implementation of these techniques involves additional computational overheads.

In 2009, Cuckoo Search (CS) as a highly potential meta-heuristic algorithm was proposed by Xin-She Yang of Cambridge University and Suash Deb of C.V. Raman College of Engineering. Recent work [35,36] has found that the CS algorithm is dominant in minimizing localization error due to the characteristics of few parameters, being easy to realize, far more efficient global search ability than existing algorithms such as GA and PSO [37]. However, the CS algorithm exhibits slow convergence rate, which makes it require more resource to achieve the certain accuracy.

Motivated by the above observations, in this paper, we propose a new modified CS algorithm to obtain positioning results with high precision and rapid convergence. The proposed CS algorithm adopts the modified step size to enable the population to approach global optimal solution rapidly. In addition, to enhance population diversity and thereby avoid local convergence, the fitness of each solution is employed to build mutation probability that determines the chance of it being found and replaced by new random solution. Finally, the modified CS algorithm restricts the population in the certain range so that it can prevent the energy consumption caused by insignificant search. The modified CS algorithm is studied on the effects of parameters like anchor density, node density and communication range with respect to average localization error and localization success ratio. Experimental results prove that the modified CS algorithm can not only increase convergence rate but also reduce average localization error compared with standard CS algorithm and PSO algorithm.

The rest of the paper is organized as follows: Section 2 provides a description of standard CS algorithm; Section 3 gives an introduction about the modified CS algorithm; WSN node localization process based on the modified CS approach is explained in Section 4; simulation results and localization performance analysis are given in Section 5; Section 6 gives the conclusion of the paper.

## 2. Standard Cuckoo Search Algorithm

Cuckoo Search (called CS), as one of the latest nature-inspired search algorithm, was proposed in 2009 by Xin-She Yang of Cambridge University and Suash Deb of C.V. Raman College of Engineering. CS is based on the brood parasitism of some cuckoo species. To apply CS as an optimization tool, Yang and Deb described the CS by three ideal rules [38]:

(1) Each cuckoo lays one egg at a time, and dumps its egg in a randomly chosen nest;
(2) The best nests with high-quality eggs will be carried over to the next generations;
(3) The number of available host nests is fixed, and the egg laid by a cuckoo is discovered by the host bird with a probability Pa∈[0,1]. In this case, the host bird can either get rid of the egg, or simply abandon the nest and build a completely new nest.

As a further approximation, this last assumption can be approximated by a fraction Pa of the n host nests are replaced by new nests (new random solutions).

For the implementation point of view, we can generate new solutions (cuckoos) by two methods: $Le^{\prime }vy$ flight and a fraction Pa of the n host nests are replaced by new random solutions.

Firstly, the $Le^{\prime }vy$ flight process, which has previously been used in search algorithms, is a random walk that is characterized by a series of instantaneous jumps chosen from a probability density function which has a power law tail. When generating new solutions $x^{\left(t+1\right)}$ for, say, a cuckoo i, a $Le^{\prime }vy$ flight is performed by using Equation (1):

(1) $${x_{i}}^{\left(t+1\right)}={x_{i}}^{\left(t\right)}+\alpha ⊕Le^{\prime }vy(\lambda )$$

where α>0 is the step size which should be related to the scales of the problem of interests. In most cases, α=ο(L/10) where L is the characteristic scale of the problem of interest. The above Equation (1) is essentially the stochastic equation for a random walk. In general, a random walk is a Markov chain whose next status/location only depends on the current location (the first term in the above Equation (1)) and the transition probability (the second term in the above Equation (1)). The product ⊕ means entry-wise multiplications. This entry-wise product is similar to those used in PSO, but here the random walk via $Le^{\prime }vy$ flight is more efficient in exploring the search space, as its step length is much longer in the long run.

The step length of random walk formed by $Le^{\prime }vy$ flight obeys the $Le^{\prime }vy$ distribution which has an infinite variance with an infinite mean as follows:

(2) $$Le^{\prime }vy∼u=t^{-\lambda },(1<\lambda \leq 3)$$

where λ is a constant, and the step length can be calculated mathematically using the following equation:

(3) Le′vy(λ)=u|v|1λ

where *u* and *v* are drawn from normal distributions as followings:

(4) $$u∼N(0,{\sigma_{u}}^{2}),v∼N(0,{\sigma_{v}}^{2})$$

where:

(5) σu={Γ(1+λ)sin(πλ2)Γ[(1+λ)2]λ2(λ−1)2}1λ,σv=1

Briefly speaking, the step length essentially forms a random walk process with a power-law step-length distribution with a heavy tail. Some of the new solutions should be generated by $Le^{\prime }vy$ walk around the best solution obtained so far, thus this will speed up the local search.

In addition, a fraction Pa of the solutions will be replaced by new random solutions whose locations should be far enough from the current best solution. This will make sure that the system will not be trapped in a local optimum.

## 3. Modified Cuckoo Search (CS) Algorithm

In standard CS algorithm, the efficiency of searching global best solution is mainly guided by step size control factor α and mutation probability Pa. Because the parameters are kept constant, i.e., α=0.01, Pa=0.25, this can result in decreasing location precision which will affect the practical value of WSN applications and reducing convergence rate which will shorten the life time of sensor nodes with limited energy resource because of a large number of iterations and long computational time. Although some improvement schemes based on standard CS algorithm have been proposed to meet specific requirements, but they are not greatly significant for WSN localization [39,40]. Therefore, to solve the current situation, this paper tries to take strategies from the below aspects discussed to propose an effective CS algorithm for WSN node localization.

One aspect is the modification about step size α. As is shown in $Le^{\prime }vy$ flight Equation (1) in Section 2, if the value of α is set to be too small, the population in the search procedure will focus on a small field around the current local best solution found in the past search. In other words, it is insignificant for generating diverse solutions to explore the search space on the global scale. In the end, global best solution cannot be found unless there is a large number of iterations which may result in relatively high computational consumption for energy-limited WSN. If the value of α is set to be too large, the according step length will make the new solutions generated too far away from the current local best solution. Finally, it is not guaranteed to converge to global optimal solution. Consequently, selecting a proper step size is very important to find the global best solution as efficiently as possible.

Based on the above analysis, we propose that step size α is modified using the Equation (6).

(6) $$\alpha =\alpha_{\text{max}}-(N\_iter/N\_itertotal)\times (\alpha_{\text{max}}-\alpha_{\text{min}})$$

where $\alpha_{\text{max}}$ and $\alpha_{\text{min}}$ denote the maximum and minimum of step size, respectively; N_iter and N_itertotal denote the current iteration number and total number of iterations, respectively.

As expressed in the Equation (6), step size α decreases with the increasing of iteration numbers. At the beginning of iterations, step size takes the maximum which can promote global search, then local search is intensified gradually by decreasing step size. The reason for this modification is that the population tends to global optimal solution gradually with the increasing of iterations.

The other aspect, we employ the fitness of solutions to build the mutation probability Pa which can enhance population diversity and avoid local convergence. If mutation probability Pa is set to be too large, the convergence rate cannot be accelerated in time; if mutation probability Pa is set to be too small, then the accuracy decreases and a large number of iterations are caused in order to obtain good performance. Therefore, the setting of parameter Pa should be neither too large nor too small.

According to the standard CS algorithm, mutation probability Pa is associated with the fitness of the solution, which can simply be proportional to the value of an objective function. A nest having higher fitness value which is closer to current global optimum than the other nest that is far away, has a higher probability to be chosen as the member of populations of next generation, in other words, it can be more hard to be found and replaced by new random solution, that is, the value of mutation probability Pa can be relatively smaller, and vice versa.

The above analysis is taken as the basis for the following adjustment about mutation probability Pa:

(7) $$\begin{array}{cc} Pa\left(j\right)=\left\{ \begin{array}{l} Pa_{\text{min}}+(Pa_{\text{max}}-Pa_{\text{min}})\times K,K<1\\ \frac{Pa_{\text{max}}}{N\_iter},otherwise \end{array}\right. & j=1,2,3,\ldots n \end{array}$$

where $K=fitness(j)-f_{\text{min}}$, which depends on the current quality of jth solution; fitness(j) and $f_{\text{min}}$ represent the current fitness of jth solution and current global optimal fitness of the population, respectively; $Pa_{\text{max}}$ and $Pa_{\text{min}}$ represent the maximum and minimum of mutation probability Pa, respectively.

From the Equation (7), it can be seen that the fitness of the solution is adopted to adjust Pa. If K≤1, it means that the current quality for jth solution is close to current global optimum. It is suggested that mutation probability Pa should be proportional to K which represents the current quality of jth solution. The higher the quality is, the smaller mutation probability Pa is. In contrast, if K>1, it means that the current quality of jth solution is far away from current global optimum. We propose that mutation probability Pa vary inversely with iteration times because the population will approach the global optimum as the iteration number increases.

Besides that, the population is restricted in the certain range so that it can prevent the energy consumption caused by meaningless search. That is to say, when the solutions are outside the range, we use the range maximum or minimum to replace them. A modified CS algorithm is proposed herein relying on the above several aspects to find the global optimum as efficiently as possible. The detailed steps about realization of the modified CS algorithm are described in Algorithm 1.
**Algorithm 1.** Modified CS algorithm1. Begin2. Generate initial population of n nests (solutions) $x_{i}$, i = 1, 2, …, n3. Define objective function f(x); x = (x_1_, x_2_, …, x_d_);4. Set the range of α and Pa: $\alpha_{\text{min}}$, $\alpha_{\text{max}}$, $Pa_{\text{min}}$, $Pa_{\text{max}}$5. Set the range of the nest(solution): $X_{\text{min}}$, $X_{\text{max}}$6. Set the maximum number of iterations: N_itertotal7. For all $x_{i}$ do8. Calculate the fitness $F_{i}=f(x_{i})$9. End For10. N_iter = 111. While (N_iter < N_itertotal) do12. For all $x_{i}$ do13. Compute the step size for $Le^{\prime }vy$ flight using Equation (6)14. Generate a new cuckoo ($x_{j}$) from the nest $x_{i}$ randomly by taking Lévy flight15. If ($x_{j}>X_{\text{max}}$) then16. $x_{j}\leftarrow X_{\text{max}}$17. End If18. If ($x_{j}<X_{\text{min}}$) then19. $x_{j}\leftarrow X_{\text{min}}$20. End If21. Calculate the fitness $F_{j}=f(x_{j})$22. Choose a random nest ($x_{k}$) among n nest randomly23. If ($F_{j}>F_{k}$) then24. $x_{j}\leftarrow x_{k}$25. $F_{j}\leftarrow F_{k}$26. End If27. End For28. Keep the current global optimal fitness: $f_{\text{min}}$29. Compute the probability Pa using Equation (7)30. A fraction (Pa) of worse nests abandoned and new ones/solutions are built/generated correspondingly31. For all the nests (say, $x_{i}$) to be built/generated do32. If ($x_{i}>X_{\text{max}}$) then33. $x_{i}\leftarrow X_{\text{max}}$34. End If35. If ($x_{i}<X_{\text{min}}$) then36. $x_{i}\leftarrow X_{\text{min}}$37. End If38. Calculate the fitness $F_{i}=f(x_{i})$ and evaluate its quality/fitness $F_{i}$39. Keep best solutions (or nests with quality solutions)40. End For41. Rank all the solutions and find the current best42. End While43. End


## 4. WSN Node Localization Process Based on the Modified CS Algorithm

**Figure 2:**
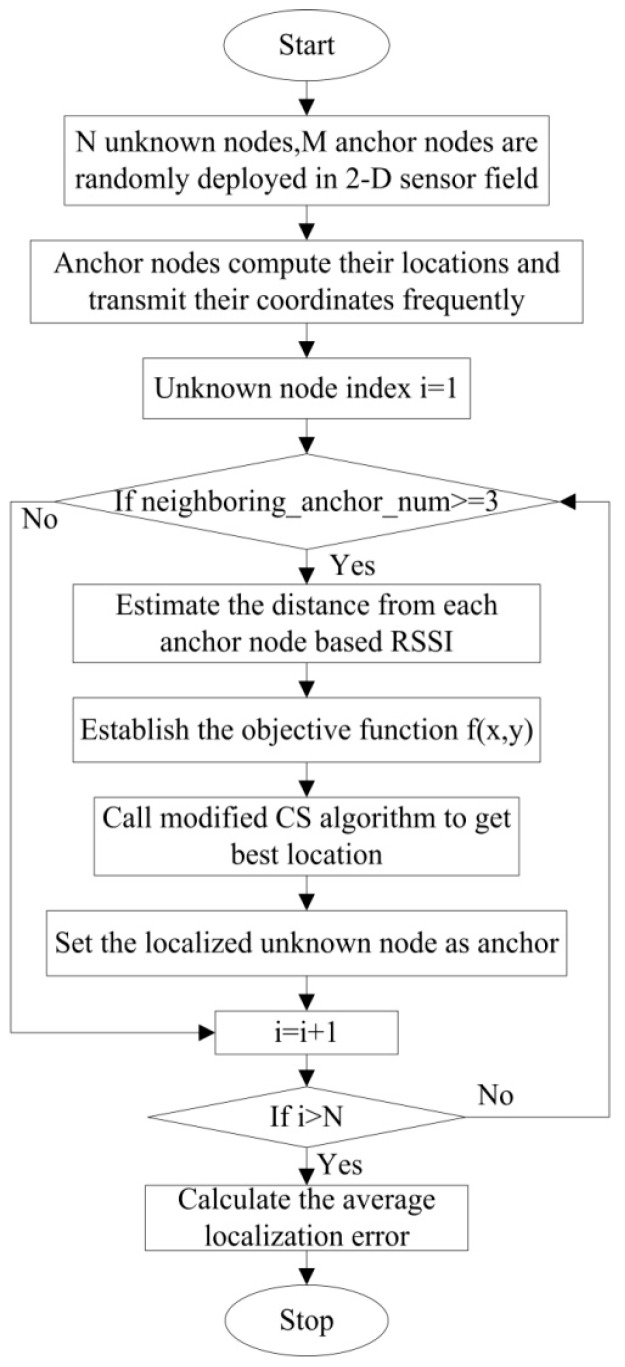
Flowchart of WSN node localization process based on modified CS algorithm.

The objective of WSN localization is to estimate the coordinates of N unknown nodes based on M anchor nodes. Assuming that all sensor nodes are deployed in a two-dimensional sensor field. The WSN node localization process based on modified CS algorithm is shown in the Figure 2.

As presented in Figure 2, estimating the locations of unknown nodes mainly includes the following steps:

Step 1: M anchor nodes and N unknown nodes are randomly deployed in a sensor field. The communication range for each sensor node is set to R.
Step 2: Anchor nodes broadcast their locations frequently.
Step 3: If ith unknown node has three or more than three anchors within communication range, it will be considered to be localizable. Owing to RSSI (received signal strength indicator) of simple implementation and low cost in hardware under actual deployment scenario, in this paper, we assume that distance measurement between neighboring nodes is realized based on RSSI that can then be transferred into equivalent distances for positioning intersection. However, RSSI-based ranging is usually affected by multi-path and obstacles blocking, which can be modeled as log-normal shadowing. The result of the log-normal model is that RSSI-based distance estimates have ranging error which follows a zero-mean Gaussian distribution with variance $\sigma^{2}$. In addition, the standard deviation of ranging error is proportional to the actual distance $d_{ij}$ between node $\left(x_{i},y_{i}\right)$ and $\left(x_{j},y_{j}\right)$ [8,10], as shown in the Equation (8).
  (8) $$\sigma^{2}=\gamma^{2}\times {d_{ij}}^{2}$$
  where noise factor γ is set to 0.1 in the experiment, $d_{ij}$ is the real distance between the unknown node $\left(x_{i},y_{i}\right)$ and jth anchor $\left(x_{j},y_{j}\right)$ within communication range, such as the Equation (9).
  (9) $$d_{ij}=\sqrt{{\left(x_{i}-x_{j}\right)}^{2}+{\left(y_{i}-y_{j}\right)}^{2}}$$
  The measured distance ${d_{ij}}^{\prime }$ between unknown node $\left(x_{i},y_{i}\right)$ and jth anchor node $\left(x_{j},y_{j}\right)$ is modeled using Equation (10).
  (10) $${d_{ij}}^{\prime }=d_{ij}+N_{ij}$$
  where $N_{ij}$ represents ranging error between unknown node $\left(x_{i},y_{i}\right)$ and its neighboring anchor node $\left(x_{j},y_{j}\right)$. Meanwhile in this paper, we assume that the random ranging error $N_{ij}$ different from each other, that is to say, if (i,j)≠(k,p), $N_{ij}\neq N_{kp}$.

Step 4: Establishing the objective function $f\left(x_{i},y_{i}\right)$. The objective function representing mean of square of ranging error between the unknown node and anchors, is defined as Equation (11):
  (11) $$f(x_{i},y_{i})=\frac{1}{m}\sum_{j=1}^{m}{\left(d_{ij}-{d_{ij}}^{\prime }\right)}^{2}$$
  where m(m≥3) is the number of anchor nodes within communication range, the unknown node can estimate its coordinate by running the modified CS algorithm. That is, when the objective function is minimized, corresponding $\left(x_{i},y_{i}\right)$ is the position of ith unknown node.
Step 5: The unknown nodes that get localized will act as anchors in the next iteration, thus the number of anchors increase along with the iteration progress.
Step 6: Step 2~Step 5 are conducted repeatedly until no unknown nodes can be localized or termination conditions are reached.
Step 7: Computing the average localization error. The average localization error is defined as the average Euclidean distance between the real and estimated locations of sensor nodes, thus average localization error can be calculated via the following Equation (12).
  (12) $$Average\;localization\;error=\frac{\sum_{i=1}^{NL}\sqrt{{\left(X_{i}-x_{i}\right)}^{2}+{\left(Y_{i}-y_{i}\right)}^{2}}}{NL}$$
  where NL is the number of localized node, $\left(x_{i},y_{i}\right)$ is the computed node location and $\left(X_{i},Y_{i}\right)$ is the actual node location. The smaller average localization error, the better the localization performance.

## 5. Simulation Experiments and Performance Evaluation

In this section, all the simulation experiments are carried out by using MATLAB. The performance of the modified CS algorithm is assessed from the effects of several factors, such as anchor density, communication range, node density in terms of average localization error, localization success ratio and a comparative study with standard CS algorithm and PSO algorithm is conducted to realize the same localization task using the same network deployment. Average localization error is computed using Equation (12) in Section 4.

Localization success ratio, which is defined as the number of unknown nodes which successfully acquire their locations over the total number of sensor nodes whose locations is unknown, is calculated using the following Equation (13). The higher localization success ratio is, the better the localization performance is.

(13) $$Localization\;success\;ratio=\left(\frac{Number\;of\;unknown\;nodes\;localized}{Total\;number\;of\;unknown\;nodes}\right)\times 100\%$$

### 5.1. Simulation Setup

All the sensor nodes are randomly deployed in 100 × 100 m^2^ two-dimensional sensor area using a continuous uniform distribution pseudo-random generator. The total number of sensor nodes, namely node density, is set to be 100, 200, 300 and 400. Anchor nodes account for 10%~60% of all sensor nodes. Anchor nodes are deployed randomly. It is assumed that there is no localization error for anchor nodes. Communication range for all sensor nodes is set to be 10 m~50 m. To eliminate the effects of randomness of topology generation and bio-inspired algorithm, each data point is averaged over 10 different test network and each result for a kind of network topology is averaged by running 30 times repeatedly. Extensive experiments have been conducted to design the parameters in the proposed CS algorithm and Experimental results demonstrate that higher localization precision with less iterations can be achieved when the parameters setting of the proposed CS algorithm are as follows, $\alpha_{\text{min}}$ and $\alpha_{\text{max}}$ are set to 0.9 and 1.0 respectively, $Pa_{\text{min}}$ and $Pa_{\text{max}}$ are set to 0.05 and 0.25 respectively. To prevent the search length over the sensor field, the boundary of sensor field is set to be the range of each nest. Thus, in 100 × 100 m^2^ two-dimensional sensor area, $X_{\text{min}}$ and $X_{\text{max}}$ are set to 0 and 100 respectively. As standard CS algorithm, the value of nest number is 25. The iteration threshold for each algorithm is 100 times.

### 5.2. Experimental Results and Performance Analysis

#### 5.2.1. The Effect of Anchor Density

Anchor density is an important parameter affecting the localization performance and cost for WSN. In this subsection, the effects of anchor density on localization performance are evaluated. Anchor ratio is set to be 10%, 20%, 30%, 40%, 50% and 60% of all sensor nodes. The communication range of each sensor node is 25 m.

**Figure 3:**
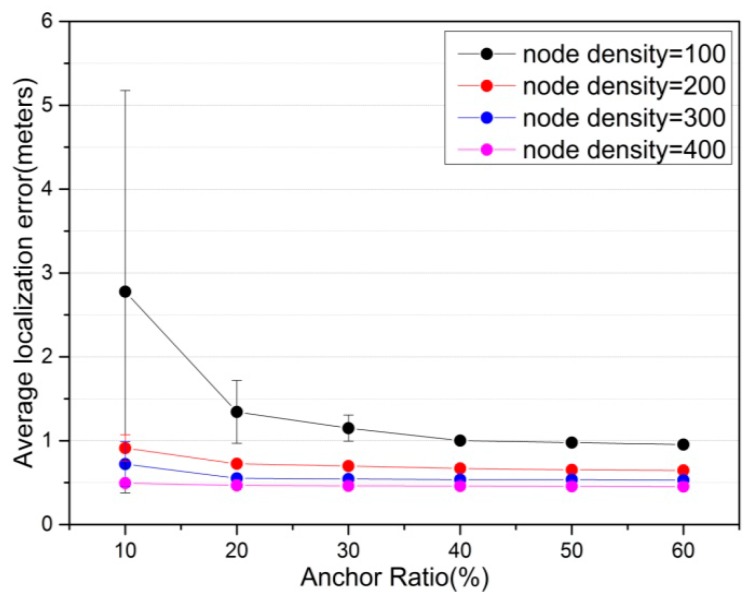
The effect of anchor ratio on average localization error. In addition, error bar represents 95% confidence interval of average localization error.

In Figure 3, using the proposed CS algorithm, the average localization error and confidence interval of location error are evaluated by varying anchor ratio in the network for different node density. The confidence interval represents a range estimate of average localization error. It can be clearly observed that when the anchor ratio in the network increases from 10% to 40%, the localization accuracy improves significantly. Because with the increasing number of anchor nodes, the number of unknown nodes that can realize localization based on original anchor nodes (the nodes are localized by BDS/GPS receivers or manual deployment) within their communication range increases. However, when anchor ratio continues to expand, the effects on average localization error become insignificant. It means that sometimes there is no need to increase the number of anchor nodes requiring the extra specific hardware which may be expensive. In addition, as node density increases, average localization error decreases correspondingly. This is because when node density increases, the number of anchor nodes within communication range increases accordingly.

The simulation results in Figure 3 show that the confidence interval is widen obviously when anchor ratio is 10% and node density is 100. This may be attributed to the fact that there are very few original anchors within communication range for many unknown nodes and error propagation occurs between sensor nodes, which has a negative impact on the average localization error.

**Figure 4:**
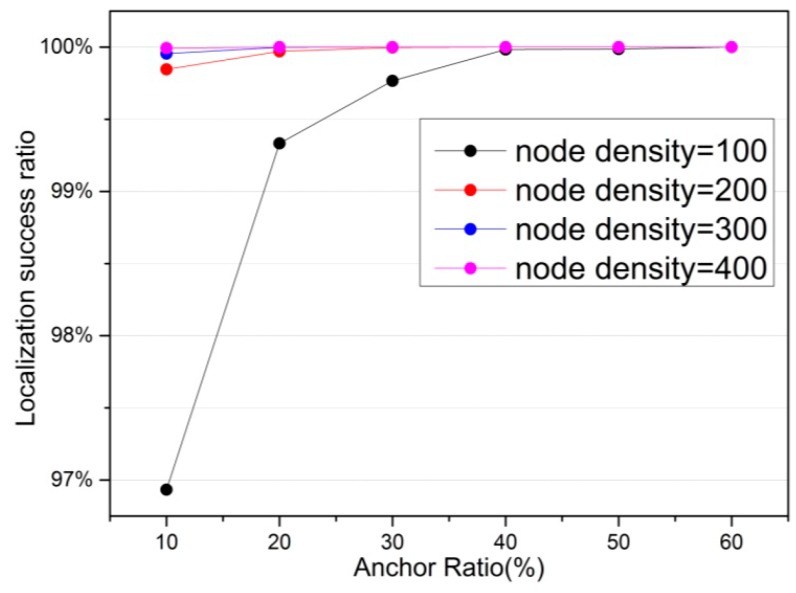
The effect of anchor ratio on localization success ratio.

Figure 4 illustrates the variation of localization success ratio under different anchor ratio and node density in the network. It demonstrates the fact that when anchor ratio increases, localization success ratio also increases. In addition, it is noted that 100% localization success ratio is achieved under the certain anchor ratio. In addition, expanding the node density improves localization success ratio correspondingly. The simulation results above show that it is necessary to deploy proper number of anchor nodes and node density for the efficient localization accuracy and localization success ratio.

#### 5.2.2. The Effect of Communication Range

**Figure 5:**
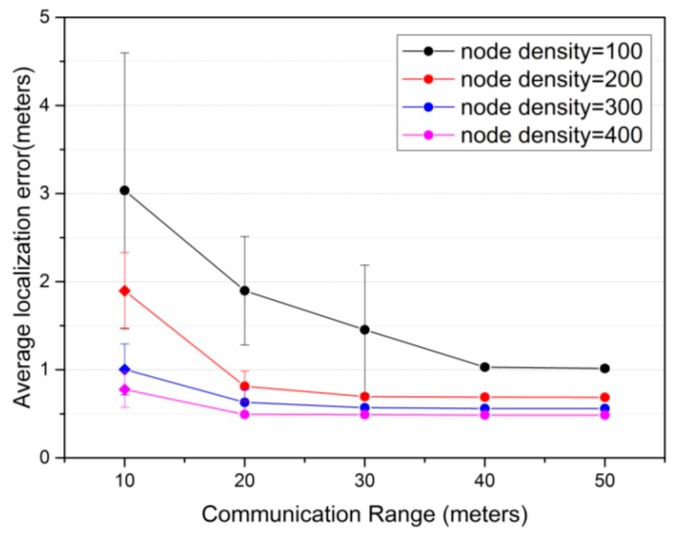
The effect of communication range on average localization error. In addition, the error bar denotes 95% confidence interval of average localization error.

**Figure 6:**
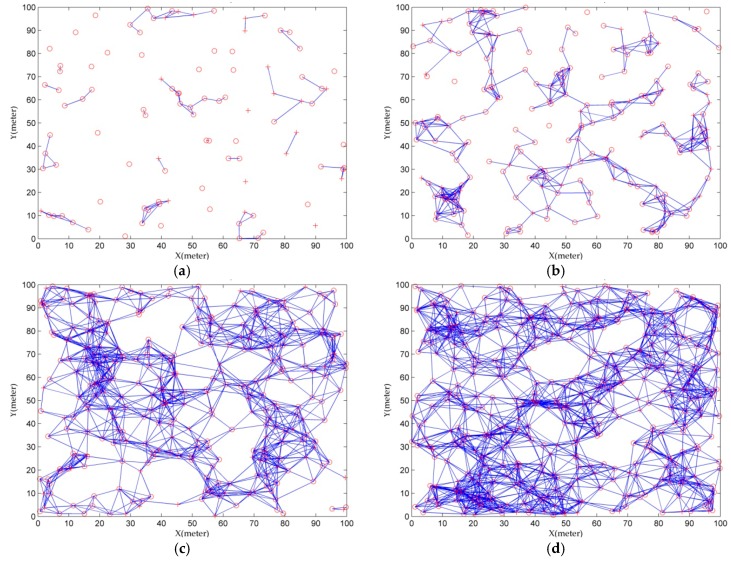
Example of network topology under different node density, (**a**) 100; (**b**) 200; (**c**) 300; (**d**) 400 when communication range is 10 m and the number of anchor nodes (+) is 20% of node density. In addition, the (∘) represents unknown nodes, the (−) represents the communication connectivity between sensor nodes.

Communication range is another important parameter determining localization performance and energy consumption of sensor nodes. The impact of communication range on the proposed CS algorithm in terms of average localization error under different node density is shown in Figure 5. The number of anchor nodes is set as 20% of all sensor nodes. The variation of communication range is from 10 m to 50 m. From Figure 5, we can observe that if the communication range is smaller than 20 m, the average localization error is slightly larger. This is because the network is not connected for many nodes, such as the example of network topology in Figure 6. As communication range increases, average localization error decreases greatly. This is due to the fact that there is more anchor information available for computing the location of unknown nodes. Even so, when communication range increases to a certain value, average localization error drops slightly. The localization accuracy also depends on the node density that is introduced in the above. It can be observed that the average localization error is decreased gradually with respect to the increasing of node density. This is due to the fact that when node density increases, the network connectivity between sensor nodes becomes high and the number of anchor nodes available within communication range increases, as the example presented in Figure 6.

**Figure 7:**
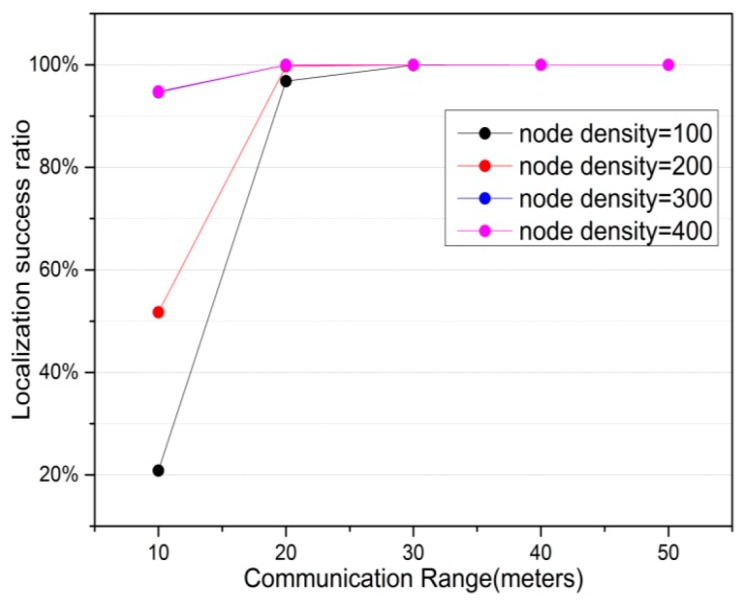
The effect of communication range on localization success ratio.

Figure 7 presents the variation of localization success ratio under the impact of communication range for different node density. In Figure 7, it is remarkable that when the communication range is smallest (that is 10 m) and node density is 100, localization success ratio is around 20.8%. This is because there are few anchors for many unknown nodes within the communication range, and the network connectivity between sensor nodes is poor as the example illustrated in Figure 6a. When the communication range increases, localization success ratio is improved evidently, because as mentioned above, there are more anchor information available in communication range. Therefore it is more easily to locate the unknown nodes. When the communication range increases to a certain degree, the localization success rate will reach the highest, 100%. In addition, when node density increases, localization success ratio is also increased correspondingly. Figure 5 and Figure 7 indicate that in practical scenario the high localization success ratio and small average localization error can be achieved by using the proposed CS algorithm. The choice of communication range depends on the localization requirements, such as localization accuracy, localization success ratio and the energy constraint of sensor nodes.

#### 5.2.3. Comparison with Standard CS and PSO Algorithm

**Figure 8:**
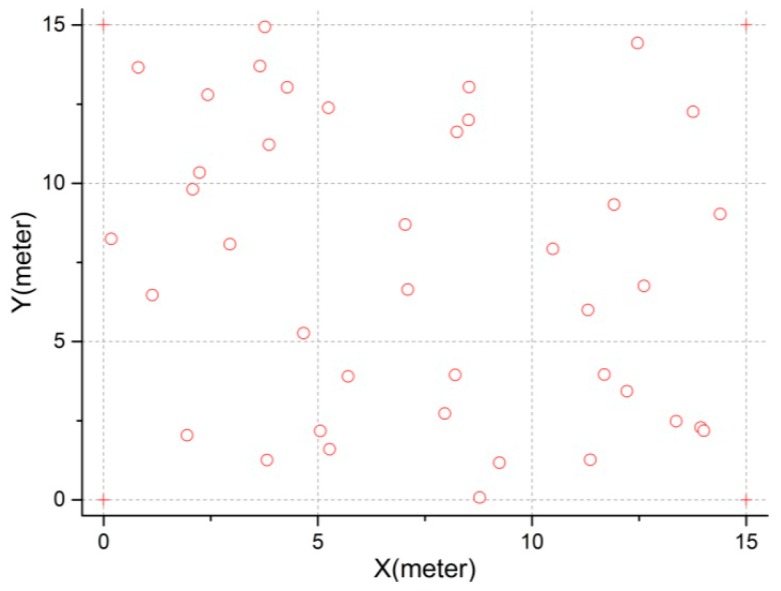
Deployment diagram of 44 sensor nodes with 4 anchor nodes (+) and 40 unknown nodes (∘) in 15 × 15 m^2^ sensor field.

**Figure 9:**
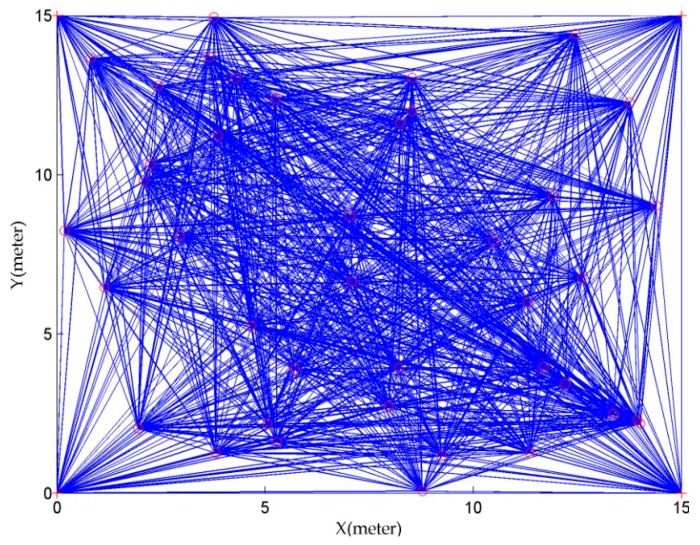
Network topology of sensor nodes, the (∘) represents unknown nodes, the (+) represents anchor nodes and the (−) represents the communication connectivity between sensor nodes.

To evaluate the effectiveness of modified CS algorithm, a comparative study was carried out with standard CS algorithm in this subsection. The parameters for standard CS algorithm are set (according to the literature [38]) as follows, the number of nests is 25, the mutation probability Pa is 0.25, the step size α is 0.01. Besides, to prevent the insignificant search over the sensor field, $X_{\text{min}}$ and $X_{\text{max}}$ for the modified CS algorithm are set to 0 and 15 respectively. The network deployment is shown in Figure 8. 40 unknown nodes and 4 anchors are deployed in a 15 × 15 m^2^ sensor field. Anchors are specially deployed in the four corner of sensor area to avoid the collinear problem like the literature [8]. Their coordinates are (0, 0), (0, 15), (15, 0) and (15, 15). In addition, it is assumed that each sensor node can communicate with every other sensor node in the sensor field, as shown the network connectivity in Figure 9.

It has been reported in previous work [30,41,42] that the PSO algorithm provides much better performance than Simulated Annealing (SA), Genetic Algorithm (GA), BBO variants and traditional mathematical optimization methods in terms of computational complexity and location accuracy. Thus, localization performance of the modified CS algorithm is also compared with PSO algorithm. The parameters of PSO algorithm are set (according to the literature [29,30]) as follows:

(1) Population size = 20;
(2) Acceleration constants c1 = c2 = 2;
(3) Inertia weight w = 0.7;
(4) Limits on particle velocity: $V_{\text{max}}$ = 15 m/s, $V_{\text{min}}$ = –15 m/s.

**Figure 10:**
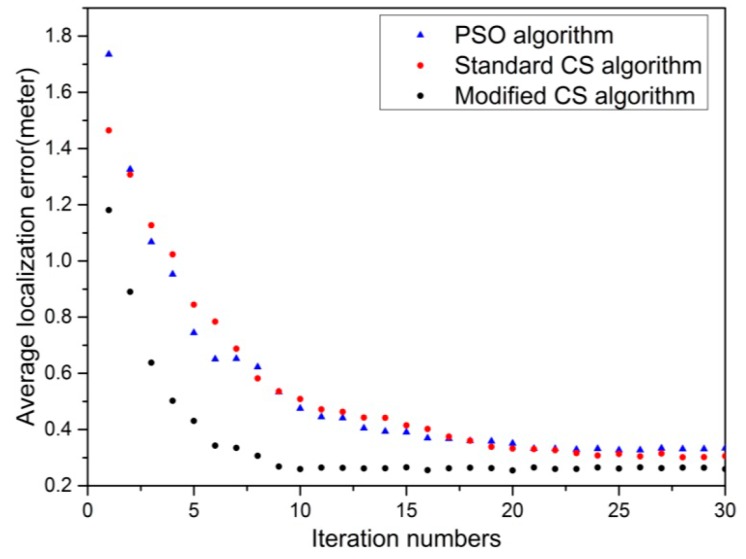
Comparison with standard CS and PSO algorithm.

To assess the convergence speed to a minimum average localization error, each algorithm is conducted for thirty iterations. The average localization error with respect to iterations is shown in Figure 10, in which each data point is the average of the results of thirty independent experiments. This is due to the fact that standard CS, PSO and modified CS algorithm are stochastic algorithms; the same result cannot be obtained in all the runs even with the same network deployment. Taking into account the characteristics of stochastic algorithm, it is assumed that convergence is reached if the fluctuation of convergence curve with respect to average localization error is less than 0.01 m.

Figure 10 reflects that as iterations proceed, all algorithms gradually converge to a minimum average localization error at different speeds. The convergence speed reflects the efficiency of the algorithm to find optimal locations. In addition, the algorithm with less iteration requires less resource, which makes it more suitable for WSN. The average localization error of the modified CS algorithm decreases more rapidly than that of PSO algorithm and standard CS algorithm during the first six iterations and the modified CS algorithm converges approximately in the tenth iteration with the average localization error of 0.259 m while the standard CS algorithm takes about twenty-four iterations to reach the convergence with average localization error of 0.307 m. In addition, the PSO algorithm seems to convergence at approximately twenty-second iteration with the average localization error of 0.332 m. From the results, it can be seen that the modified CS algorithm can reach the same localization accuracy as standard CS algorithm and PSO algorithm with much fewer iterations. It proves that the modifications of the proposed CS algorithm in increasing convergence speed and location accuracy are observable and effective. This means that it is possible to adopt the proposed CS algorithm in optimizing WSN node localization instead of standard CS algorithm and PSO algorithm.

## 6. Conclusions

In this paper, we propose a modified CS algorithm for optimizing node localization in WSN. The algorithm adopts the modified step size to enable the population to approach global optimal solution rapidly, and the fitness of each solution is employed to build mutation probability to avoid local convergence. In addition, to prevent the energy consumption caused by insignificant search, the approach restricts the population in the certain range. Extensive experiments have been performed to study the impacts of several factors like anchor density, node density and communication range on the proposed algorithm with respect to average localization error and localization success ratio. This provides the basis for optimizing node localization using the proposed algorithm in practical WSN applications. Additionally, a comparative study has been conducted and experimental results prove that when compared with standard CS and PSO algorithm, the modified CS algorithm performs better in terms of reducing average localization error and increasing the convergence rate, which is favorable to reduce computational consumption and thus prolong the lifetime of sensor nodes. The effectiveness of the modified CS algorithm was verified through simulation results. In the future, we will focus on designing an experiment system to test the modified CS algorithm in practical applications.
